# New single nucleotide polymorphism G5508A in the *SEPT12* gene may be associated with idiopathic male infertility in Iranian men

**Published:** 2015-08

**Authors:** Maryam Shahhoseini, Mahnaz Azad, Marjan Sabbaghian, Maryam Shafipour, Mohammad Reza Akhoond, Reza Salman-Yazdi, Mohammad Ali Sadighi Gilani, Hamid Gourabi

**Affiliations:** 1 *Department of Genetics, Reproductive Biomedicine Research Center, Royan Institute for Reproductive Biomedicine, ACECR, Tehran, Iran.*; 2 *Department of Andrology, Reproductive Biomedicine Research Center, Royan Institute for Reproductive Biomedicine, ACECR, Tehran, Iran.*; 3 *Department of Epidemiology and Reproductive Health, Reproductive Biomedicine Research Center, Royan Institute for Reproductive Biomedicine, ACECR, Tehran, Iran.*

**Keywords:** *Male infertility*, *Polymorphism*, *SEPT12*, *Septin*, *Spermatogenesis*

## Abstract

**Background:**

Male infertility is a multifactorial disorder, which affects approximately 10% of couples at childbearing age with substantial clinical and social impact. Genetic factors are associated with the susceptibility to spermatogenic impairment in humans. Recently, *SEPT12* is reported as a critical gene for spermatogenesis. This gene encodes a testis specific member of Septin proteins, a family of polymerizing GTP-binding proteins. *SEPT12* in association with other Septins is an essential annulus component in mature sperm. So, it is hypothesized that genetic alterations of *SEPT12* may be concerned in male infertility.

**Objective:**

The objective of this research is exploration of new single nucleotide polymorphism G5508A in the *SEPT12* gene association with idiopathic male infertility in Iranian men.

**Materials and Methods:**

In this case control study, 67 infertile men and 100 normal controls were analyzed for genetic alterations in the active site coding region of *SEPT12*, using polymerase chain reaction sequencing technique. Fisher exact test was used for statistical analysis and p<0.05 was considered as statistically significant.

**Results:**

Genotype analysis indicated that G5508A polymorphic *SEPT12* alleles were distributed in three peaks of frequency in both control and diseases groups. Categorization of the alleles into (GG), (GA), (AA) types revealed a significant difference between infertile patients (azoospermic and asthenospermic) and normal controls (p=0.005).

**Conclusion:**

According to our finding we suggest that G5508A polymorphism in *SEPT12* gene can affect spermatogenesis in men, the opinion needs more investigation in different populations.

## Introduction

Subfertility is one of the major clinical, social and economic concern. In up to 55% of couples seeking medical attention, the male partner is diagnosed with spermatogenic failure, defined as one or more semen parameters falling below the world health organization (WHO) cut-off for normozoospermia (1). In the most severe forms of infertility with male factor azoospermia defined as complete absence of sperm from ejaculate, and asthenospermia means having defects in sperm motility (2). The etiology of spermatogenesis failure includes genetic abnormalities (3), infectious, and environmental causes (4).

Spermatogenesis is governed by the parallel and serial actions of thousands of genes, alterations in any of them or their expression may cause male infertility (5-10). In reality, only a handful of genetic alterations have clearly been shown to cause spermatogenic failure (11).

Recently, a number of reports have linked altered expression of Septin genes to a range of diseases, including male infertility (12, 13). Since 1997, 14 members of Septin proteins have been characterized in humans (SEPT1-SEPT14), some of which are tissue-specific. All of the 14 genome-mapped human Septin genes encode a highly conserved central GTP binding/hydrolysis domain which is very critical in GTPase signaling properties, as well as oligomerization between Septins and other filamentous proteins. This functional domain consists of three distinct amino acid sequence elements, G1, G3 and G4, which share sequence identity to the well-characterized Ras GTPase family (14).

Among all septins, *SEPT12* is dominantly expressed in testis tissue of adults, with an established essential role in annulus structure of mature sperm (15). Recently, it has been shown by investigators that high levels of *SEPT12* mRNA is observable exclusively in the testis of sexually mature males (human and mouse), while this mRNA was not detectable in men with sterility resulting from inability to produce mature spermatozoa. So, it has been considered that *SEPT12* is crucial for the process of spermatogenesis in mammals (16).

With this in mind, in the current study we tried to monitor genetic variations of *SEPT12* gene in an Iranian population of infertile men with non-obstructive azoospermia and asthenospermia, in order to find any relationship between genetic alterations in the *SEPT12* gene and some cases of idiopathic male infertility.

## Materials and methods

**Specimen**

The study population in this case-control report is consisted of 67 infertile men (50 azoospermia and 17 asthenospermia) who were referred to Royan Instiute, Tehran, Iran, during 2012-2013. Also, 100 normozospermic men with female factor subfertility were analyzed as control (Table I). Using power analysis and sample size (PASS, 2011), the power of the study was calculated 0.78. Patients with hypogonadotropic hypogonadism, cryptorchidism, orchitis, ejaculatory duct obstruction, and men with microdeletions of the long arm of the Y-chromosome or karyotype abnormalities were excluded from the study. The classification of men into the normozoospermic and azoospermic groups was according to WHO criteria (2). Informed consent was obtained from all individuals enrolled in the study.

Genomic DNA was extracted from peripheral blood specimens using a QIAamp DNA minikit (Qiagen Germany), according to the instructions provided by the manufacturer.

**Polymerase Chain Reaction (PCR)-Sequencing analysis**

For genetic analysis, the coding region of G1, G3 and G4 functional domains of *SEPT12* including exones 2-3 and their interval intron, and also exon 6 and its exon–intron boundaries were screened by polymerase chain reaction (PCR)-directed sequencing, using the specific primers designed by Primer3 software (Primer3.ut.ee) (Table II).

To analyze the aforementioned DNA sequences, PCR-Sequencing technique was performed with an initial denaturation at 95^°^C for 5 min, followed by 30 cycles of denaturation at 95^°^C for 45 sec, annealing at 60^°^C for 45 sec and extension at 72^°^C for 45 sec. The PCR products were confirmed by running on 1.5% agarose gel, and were applied for sequencing by an ABI 3730XL automated DNA sequencer (Macrogen, Seoul, Korea).

**Statistical analysis**

Fisher exact test was used to investigate the relationship between three sample groups (control, azoospermic and asthenospermic) and allele frequency. All statistical analysis were performed using SPSS (Statistical Package for the Social Sciences version 17.0, SPSS Inc., Chicago, IL, USA) software. A p<0.05 was considered as statistically significant.

## Results

**Figure 1:**
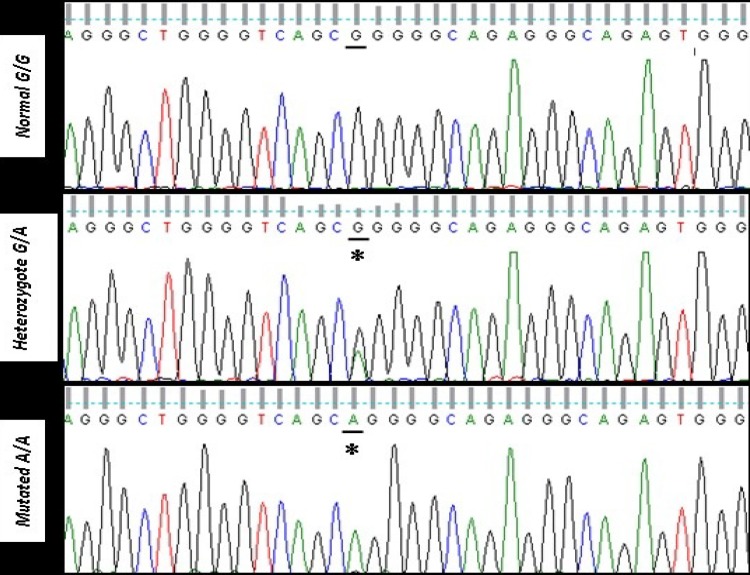
Electropherogram showing the heterozygote and homozygote DNA sequences of the *SEPT12* gene variants G→A compared with the normal-person sequence. The stars indicate location of the variations

Sequence analysis data revealed the nucleotide transition G5508A in *SEPT12* gene of the patients with respect to normal controls (Fig.1). The patients were divided into the two groups of azoospermia and asthenospermia. As indicated in Table I, among the azoospermic patients (50 individuals) 36 (72.0%) samples were normal homozygous (GG), 11 (22.0%) of them had the heterozygous (GA) mutation and 3 (6.0%) individuals were observed with both alleles mutated (AA). In asthenospermia patients, number of individuals harboring normal homozygous (GG), heterozygous (GA) and completely mutated (AA) alleles were, 11 (64.7%), 5 (29.4%) and 1 (5.9%), respectively. Statistical analysis of the data showed that the G5508A variation of *SEPT12* gene revealed significantly different genotype distributions between the normal control group and the patients groups (p=0.005). Also, the frequency of the normal G allele was significantly higher in the control group compared with the patients groups (p=0.001). However, there was no significant change between the two groups of azoospermic and asthenospermic for this allele (p=0.05). On the other side, the frequency of the A allele in normal group was significantly lower compared with the both patients groups (p=0.001). Again, no significant change was observed between the two patients groups (p=0.05).

**Table I:** Distribution of the allelic alteration of G5508A between infertile patients and normal controls

	**Normal** **n (%)**	**Azoospermia** **n (%)**	**Asthenospermia** **n (%)**	**p-value**
**Genotype-wise comparison, n (%)**				
GG	89 (89.0)a	36 (72.0)b	11 (64.7)b	
GA	11 (11.0)a	11 (22.0)ab	5 (29.4)b	0.005
AA	0 (0.0)a	3 (6.0)b	1 (5.9)b	
**Allele-wise comparison, n (%)**				
G	189 (94.5)a	83 (83.0)b	27(79.4)b	
A	11 (5.5)a	17 (17.0)b	7(20.6)b	0.001

The same letter in each rows show not significant difference between groups (p-value > 0.05) according to Fisher exact test.

**Table II:** Primer pairs used in this study

**Coding region**	**Primers (5ˈ3ˈ)**	**Product size (bp)**
G1-G3	F: GTTGATCTGGTCCCCGAAG R: TAAAACGCCCACCCTAACTG	332
G4	F: TGATGTCCTCGTCAAAGCAC R: CCCTGCTGCTGTCGTTTAT	341

bp: base pair

F: forward

R: reverse.

## Discussion

There are several reports introducing novel genes involved in spermatogenesis, in the way that their down-regulation in the testes tissues of infertile men has been identified by high throughput expression analyses (9). *SEPT12* is one of these genes which its lower expression in the testicular biopsies of infertile men is significantly related with azoospermia. In this study, we hypothesized genetic variations of *SEPT12* gene may be associated with male infertility caused by spermatogenesis failure.

The present association study revealed significantly different allele frequency of G5508A in *SEPT12* gene, among patients with azoospermia and asthenospermia with respect to control men with normal spermograms. Although the number of analyzed patients is not enough to have a final decision, our preliminary finding observed in Iranian patients suggests that this G>A alteration may play a causative role in disruption of spermatogenesis. According to the recent reports showing that some *SEPT12* SNPs may predispose men to spermatogemic failure (16-18), the current data propose that the novel G5508A polymorphism can be considered as a biomarker for idiopathic male infertility.

Today, in vitro fertilization has been established as an efficient technique to resolve infertility in couples with female factors, but it cannot be useful for severe male factors with lacks in spermatogenesis. Although testicular sperm extraction-intracytoplasmic sperm injection is now successfully used for these cases, however, it cannot benefit patients with complete failure in spermatogenesis. So, genetic diagnosis of severe male infertility is a critical subject for assisted reproductive technology.

## Conclusion

In conclusion, we suggest that the novel G5508A polymorphism in *SEPT12* gene may be associated with idiopathic male infertility in Iranian men. However, as this genetic alteration is in the intronic region, the molecular mechanism of its effectiveness is really unknown. Further study is needed to test this synonymous coding polymorphism for potential alteration of splicing between exonic elements.
